# Kidney Transplantation for Focal Segmental Glomerulosclerosis: Can We Prevent Its Recurrence? Personal Experience and Literature Review

**DOI:** 10.3390/jcm11010093

**Published:** 2021-12-24

**Authors:** Hamza Naciri Bennani, Lionel Elimby, Florian Terrec, Paolo Malvezzi, Johan Noble, Thomas Jouve, Lionel Rostaing

**Affiliations:** 1 Nephrology, Hemodialysis, Apheresis and Kidney Transplantation Department, Grenoble University Hospital, 38000 Grenoble, France; hnaciribennani@chu-grenoble.fr (H.N.B.); lelimby@chu-grenoble.fr (L.E.); fterrec@chu-grenoble.fr (F.T.); pmalvezzi@chu-grenoble.fr (P.M.); jnoble@chu-grenoble.fr (J.N.); tjouve@chu-grenoble.fr (T.J.); 2 Nephrology Department, Grenoble Alpes University, 38000 Grenoble, France

**Keywords:** plasmapheresis, rituximab, focal segmental glomerulosclerosis, recurrence, kidney transplantation, immunoadsorption

## Abstract

Background: Primary focal segmental glomerulosclerosis (FSGS) is associated with a high risk of recurrence after kidney transplantation with a major risk of graft loss despite preventive or curative treatments. Aim: to assess graft survival in FSGS kidney-transplant recipients and to compare those that had a relapse with those that had no relapse. Patients/Methods: we included 17 FSGS kidney-transplant recipients between January 2000 and January 2020, separated retrospectively into two groups (recurrences: *n* = 8 patients; no recurrences: *n* = 9 patients). FSGS recurrence was defined as having proteinuria of ≥3 g/g or urinary creatinine of ≥3 g/day. All patients received an induction therapy; maintenance immunosuppressive therapy at post-transplantation relied on tacrolimus/mycophenolate mofetil/steroids. In order to prevent or treat FSGS recurrence, patients received apheresis sessions plus rituximab. Results: FSGS recurrence rate was 47%. All patients that relapsed with a first graft also relapsed with subsequent grafts. Median time to recurrence was 3 (min: 1; max: 4745) days, despite rituximab/apheresis prophylaxis. Mean age was significantly lower in the relapsers (group 1) than in the non-relapsers (group 2); i.e., 47 ± 11 vs. 58 ± 9 years (*p* = 0.04). Time to progression to stage 5 chronic kidney disease (CKD) and young age at FSGS diagnosis were lower in group 1 compared to group 2; i.e., 5 (min: 1; max: 26) vs. 2 (min: 1; max: 26) years, and 16 (min: 4; max: 55) vs. 34 (min: 6; max 48) years, respectively. There was no difference between the two groups in terms of progression to CKD stage 5 on the native kidneys, averaging 7 years in both groups (*p* = 0.99). In group 1, seven patients received rituximab/apheresis prophylaxis, although this did not prevent the recurrence of FSGS. Conclusion: pretransplant prophylaxis with plasmapheresis/rituximab did not appear to reduce the risk of recurrence of primary FSGS on the graft, but could allow remission in the event of recurrence.

## 1. Introduction

Primary focal segmental glomerulosclerosis (FSGS) is a diagnosis of exclusion, because the histological lesion does not allow guiding the pathogenesis. It is a glomerular lesion characterized by the presence of sclerotic and hyaline lesions, which may be associated with deposits of immunoglobulin M (Ig M) and the C3 complement fraction [1]. The incidence of primary FSGS has increased, and accounts for 20–25% of adult patients undergoing biopsy for evaluation of idiopathic GN [2,3,4]. It is manifested by nephrotic proteinuria > 3 g/day in more than 70% of cases, often relatively sudden. Hypertension, microscopic hematuria, and renal insufficiency are seen in 30–45% of cases at presentation [3].

Whether primary, secondary, or genetic, FSGS progresses to chronic kidney disease (CKD); kidney transplantation is then the treatment of choice in stage 5 CKD. Primary FSGS is associated with a high risk of recurrence in a graft after kidney transplantation, reaching 30% after a first transplant and 80–100% after a second kidney transplant [5]. The precise identification of patients at risk for recurrence of primary FSGS on the graft is crucial in order to choose which type of graft to offer. Despite the significant risk of recurrence, patients with primary FSGS are often candidates for kidney transplantation. Indeed, their young age makes it difficult to contraindicate transplantation, even if it is a second or even a third transplant attempt.

Recurrence on the graft is defined by the reappearance of glomerular-type proteinuria, with or without nephrotic syndrome, and the absence of other causes that may be the origin of this proteinuria. It usually occurs very quickly, within hours or days after kidney transplantation [6]. The pathophysiology of the recurrence of primary FSGS is unknown, and the existence of a circulating factor (suPAR, CASK, anti-actin antibody, CD40) can attack podocytes quickly after unclamping [4,7,8,9,10,11,12,13,14]. Plasma exchange could eliminate this circulating permeability factor, allowing remission in more than 50% of cases [15]. In the event of recurrence, the risk of graft loss is major, and preventive or curative treatment for recurrence combines sessions of apheresis with immunosuppression (i.e., corticosteroids, calcineurin inhibitors, rituximab) without evidence-based medicine. Our single-center study aimed to assess graft survival in kidney-transplant recipients according to whether there was an FSGS relapse, and to compare characteristics of those that had a relapse with those that did not. We also performed a literature review on FSGS recurrence after kidney transplantation and its prevention.

## 2. Materials and Methods

### 2.1. Study Population

This single-center retrospective study included 17 kidney-transplant recipients with primary FSGS that attended our hospital between January 2000 and January 2020.

Diagnosis of primary FSGS was retained after kidney biopsy and in absence of other etiologies. Only a single patient whose diagnosis of FSGS was made at the age of 4 years was able to benefit from the genetic study, which came back negative.

FSGS recurrence was defined as having proteinuria of ≥3 g/g or urinary creatinine of ≥3 g/day, whatever the time of onset after transplantation. It was mandatory to eliminate other causes of early de novo proteinuria, including acute humoral rejection (donor-specific antibodies, increased serum creatinine, decreased diuresis). Kidney graft biopsy was not systematic; i.e., it was only performed when recurrence occurred late after transplantation or in the event of presence of a donor-specific alloantibody (or alloantibodies).

The time to recurrence was defined according to Cameron’s classification [16]: immediate (<48 h), early (<3 months), or late recurrence (>3 months).

Complete remission was defined as having proteinuria < 0.5 g/day for at least 2 consecutive weeks. Partial remission was defined as a reduction in proteinuria to 0.5–3 g/day or having decreased proteinuria of >50% from the baseline value for at least 2 consecutive weeks.

The main objective was to assess the survival of the renal transplant according to FSGS relapse, which was then treated by apheresis and/or rituximab.

All patients received an induction therapy before transplantation of either antithymocyte globulins or basiliximab. Maintenance immunosuppressive therapy at post-transplantation relied on tacrolimus (aiming at trough levels of 5–8 ng/mL), mycophenolate mofetil (1 g/day), and steroids (prednisone).

### 2.2. Preventing Recurrence of FSGS

#### 2.2.1. Apheresis

In order to prevent recurrence of FSGS, patients received one plasma exchange before transplantation, and then three plasma exchanges per week during the first 2 weeks post-transplantation. It was only performed in 7 patients due to a recurrence on a previous kidney graft.

However, in the event of an FSGS relapse immediate or early after transplantation the previous schedule was modified accordingly; i.e., apheresis sessions were intensified. Plasma exchanges were carried out by centrifugation using Comtec^®^ (Fresenius Kabi, Louviers, France) or Spectra Optia^®^ (BCT Terumo, Lakewood, CO, USA) separators. The plasma volume was treated as 1.5 times the plasma volume calculated using Kaplan’s formula [17]: PV = 0.065 × weight (kg) × (1 − hematocrit). The replacement fluid used was 4% albumin except in the immediate pre- and post-transplantation sessions, when fresh frozen plasma was used as the replacement fluid. Extracorporeal anticoagulation was based on regional anticoagulation using citrate with reinjection of calcium into the return venous line according to ionized calcium levels, which were maintained at 1.15–1.35 mmol/L.

After January 2016, patients that experienced a post-transplantation FSGS relapse had plasma exchanges replaced with immunoadsorption. Two Globaffin^®^ columns (Fresenius Medical Care, Bad-Homburg, Germany) were used alternately for every 500 mL of treated plasma (i.e., one was rinsed while the other filtered the plasma). The Globaffin^®^ adsorber used synthetic peptide GAM ligands that bind the constant (Fc) portion of the immunoglobulins, particularly the Ig G1, 2, and 4 subclasses. The volume of treated plasma per session was 100 mL × body weight (kg).

In group 1, one patient received pretransplant immunoadsorption sessions in preparation for an incompatible HLA kidney transplantation, and another patient received eight plasma-exchange sessions after transplantation to avoid a relapse of FSGS upon request of his nephrologist.

#### 2.2.2. Rituximab

Rituximab was used to prevent and treat the recurrence of FSGS at a dose of 375 mg/m^2^, given as one or two injections at one week apart. In the prophylactic setting, rituximab was given in a single dose. In curative treatment, rituximab was administered twice 2 weeks apart, then every 6 months depending on the clinical–biological evolution (nephrotic syndrome, serum albumin, proteinuria).

When combined with apheresis, rituximab was always given after an apheresis session.

The next post-rituximab apheresis session was performed 72 h after the rituximab injection to minimize drug elimination during apheresis.

Rituximab was administered as a pretransplant in 7 patients in group 1 due to recurrence of FSGS on a previous kidney graft, and in a single patient in group 2 in the setting of an incompatible HLA kidney with specific donor antibodies.

### 2.3. Treating FSGS Recurrence

Treatment of FSGS consisted of intensified plasma exchange or IA sessions: i.e., every other day for 2 weeks, and was then adapted to proteinuria level. Patients also received either methyl-prednisone pulses (10 mg/kg for three consecutive days) or prednisone, which was increased at 1 mg/kg/day for 3 weeks and then slowly tapered. Rituximab was also given (375 mg/m^2^ of body surface area) at the 4th and 8th apheresis sessions.

### 2.4. Collected Data

The data collected included age, body mass index (BMI), and gender. Before kidney transplantation, we determined age at diagnosis of FSGS, time to progression to CKD stage 5, previous renal transplants (dates, number, causes of graft loss), time on dialysis before transplantation (in months), magnitude of anti-HLA sensitization, and blood group. After kidney transplantation, we collected characteristics on the donor–recipient pair (HLA matching, cytomegalovirus-CMV-serostatus), time to recurrence of FSGS, induction and maintenance immunosuppressive treatments, and the use of renin–angiotensin system blockers. In addition, at regular intervals post-transplantation, we collected the following data: serum creatinine, estimated glomerular-filtration rate (GFR) (mL/min/1.73 m^2^), albuminemia, serum protein, proteinuria, IgG, IgA and IgM, renal-allograft histology, infectious complications (bacterial or viral; i.e., CMV, BK virus, Epstein–Barr virus, and DNAemia).

The study was conducted according to the guidelines of the Declaration of Helsinki and approved by CNIL (French national commission for data protection; approval number 1987785v0). The biobank collection number is BRIF BB-0033-00069.

All data are available upon request to the corresponding author.

### 2.5. Statistical Analyses

Quantitative data are presented as means ± standard deviations (SD) or medians and quartiles (Q1–Q3). Qualitative data are presented as absolute numbers with percentages. Differences were calculated using the chi-squared or Fisher’s exact test for categorical data, Student’s test and ANOVA were used for normally distributed numeric data variables, and the Mann-Whitney-U and Kruskal-Wallis tests for non-Gaussian variables.

A two-sided *p*-value of <0.05 was considered statistically significant. Analyses were conducted using R statistical software (version 4.1.2, Bell Laboratories, Holmdel, NJ, USA).

## 3. Results

### 3.1. Study Population 

We retrospectively separated the patients into two groups according to whether or not they had FSGS recurrence; i.e., FSGS recurrence (group 1: 8 patients), and no FSGS recurrence (group 2: 9 patients). Of the eight kidney-transplant recipients that had FSGS relapse, 75% had an early relapse. Median time to recurrence was 3 (min: 1; max: 4745) days. The mean age was significantly lower in group 1 than in group 2; i.e., 47 ± 11 years vs. 58 ± 9 years (*p* = 0.04). The age at diagnosis of FSGS was also lower in group 1 than in group 2; i.e., 16 (min: 4; max: 55) vs. 34 (min: 6; max: 48) years (*p* = 0.12). There was no difference between the two groups in terms of progression to CKD 5 on the native kidney, averaging 7 years in both groups (*p* = 0.99). The patients’ demographic criteria are listed in Table 1 and Table 2.

Overall, the rate of recurrence of primary FSGS on the kidney transplant was 47%. In addition, all patients that relapsed after a first graft also relapsed with subsequent grafts.

In group 1, four patients had undergone three kidney-transplant procedures, three patients had received a single kidney transplant, and one patient had undergone two kidney-transplant procedures. All group 1 patients had a FSGS relapse that resulted in loss of the previous renal graft. Taking into account only the most recent renal transplant, seven patients received prophylaxis with rituximab and apheresis at the time of surgery, although this did not prevent the recurrence of FSGS on the renal graft. In addition, in the post-transplantation setting, eight patients received rituximab: of these, seven also had apheresis sessions. After an average follow-up of 7 ± 3 years, there were two renal-graft losses with a return to dialysis, two patients remained dependent on immunoadsorption at a rate of one session every 4 weeks, and four patients were in clinical remission after treatment with apheresis and rituximab after recurrence of FSGS without immunoadsorption. The average current eGFR for group 1 patients was 59 ± 33 mL/min.

In group 2, six patients underwent one kidney transplantation, one patient had three kidney-transplant procedures, and two patients underwent two kidney-transplant procedures. The loss of previous grafts was not related to the recurrence of FSGS: graft-artery thrombosis occurred in one case, and chronic humoral rejection in the other patients. The average duration of follow-up was 7 ± 4 years. One patient received eight plasma-exchange sessions after transplantation to avoid a relapse of FSGS at request of his nephrologist, and another patient received 24 IA sessions associated with two infusions of rituximab at pretransplant in preparation for HLA-incompatible kidney transplantation, most likely also eliminating any factor responsible for FSGS. The current average eGFR for all group 2 patients was 65 ± 25 mL/min. All patients had nondetectable proteinuria. No patient lost their graft.

Patients from both groups had immediate resumption of graft function; i.e., no patient received immediate postoperative dialysis. No patients presented with any complications related to apheresis (hemodynamic tolerance, hypo- or hypercalcemia). No patient had donor-specific antibodies at the time of the transplantation.

### 3.2. Infectious Complications

In group 1, CMV DNAemia was assessed weekly for the first month, and then before each apheresis session during the maintenance period. In group 2, CMV DNAemia was assessed every 2 weeks for the first 6 months post-transplantation. All the patients except those that were donor CMV seronegative/recipient seropositive underwent CMV prophylaxis for the first three months post-transplant with valganciclovir adapted to eGFR.

If CMV, DNA level was >3 log copies/mL, treatment with valganciclovir was initiated (900 mg × 2/day) for 3 weeks and adapted to GFR. Overall, only four patients (all from group 1) presented with mild CMV reactivation.

## 4. Discussion

The rate of recurrence of primary FSGS on a renal graft was 20 to 40% for a first graft, but reached 80 to 100% recurrence on subsequent grafts if there was recurrence on the first graft [5,18]. In our case series, the rate of recurrence of primary FSGS on the kidney transplant was 47%. Kalliopi Vallianou et al. [19] also found 54% recurrence of primary FSGS (25 patients) among 46 kidney-transplant recipients; in addition, recurrence developed very soon after transplantation; i.e., the median recurrence time was 0.5 months (0.1–1). The main risk factors for FSGS recurrence were recurrence on a previous transplant, rapid progression to stage 5 CKD on native kidneys, and young age at the time of initial diagnosis [5,20,21,22]. An albuminemia level of <25 g/L at the time of diagnosis was also considered to be a risk factor for recurrence on the renal transplant [23]. In contrast, the type of donor (living vs. deceased) did not influence the risk of FSGS recurrence [19].

In our study, all patients that relapsed with a first graft also relapsed with subsequent grafts. The time to progression to stage 5 CKD and young age at diagnosis of FSGS were lower in the relapsers (group 1) compared to the non-relapsers (group 2); i.e., 5 (min: 1; max: 26) vs. 2 (min: 1; max: 26) years, and 16 (min: 4; max: 55) vs. 34 (min: 6; max: 48) years, respectively. Two of our patients received transplants from living donors.

Despite rituximab and apheresis prophylaxis, group 1 patients had FSGS recurrence after a median of 3 (min: 1; max: 4745) days, and two subsequently lost their allograft. In general, renal transplantation from a living donor allowed better graft survival compared to transplantation from a deceased donor. However, in view of the significant risk of recurrence of primary FSGS, it was not advisable to propose a kidney transplant from a living donor in the event of primary FSGS, especially if there had been recurrence on a previous graft because, to date, there is still no effective prophylaxis that can prevent FSGS recurrence [24,25].

In group 2, no patient had FSGS recurrence in the kidney transplant. The mean age in this group at FSGS diagnosis was 34 (min: 6; max: 48) years. The genetic study was not carried out in these patients. We did not find any secondary causes of FSGS, but we therefore could not rule out a genetic cause explaining absence of FSGS recurrence. Morello et al. [26], in a study published on 101 kidney transplant patients for steroid-resistant nephrotic syndrome (SRNS), after a median follow-up of 58.5 months, found a SRNS recurrence in the first renal transplant in 53.3% of patients with a nongenetic cause, and in none in those who had a genetic SRNS. They concluded that absence of a causative mutation represented the major risk factor for post-transplant recurrence in children with SRNS.

In our series, prophylaxis with rituximab and apheresis did not appear to reduce the risk of recurrence of primary FSGS. In fact, the combination of plasmapheresis and rituximab immediately before kidney transplantation in 87.5% of these patients did not prevent recurrence. However, after an average follow-up of 7 ± 3 years, this association of treatments seemed to be effective when recurrence occurred at post-transplantation; i.e., remission was induced in 50% of cases.

Prophylaxis is based on the assumption of the existence of a soluble permeability factor [4,5]. This factor has been suggested by several observations, including the development of proteinuria in rats after injection of proteins eluted from an immunoadsorption (IA) column used by FSGS-treated patients [27]. This was also suggested by: (i) the disappearance of nephrotic syndrome after transplantation of a kidney from an FSGS patient into a non-FSGS kidney recipient [7,28,29]; (ii) the occurrence of a nephrotic syndrome in a newborn of an FSGS mother [30]; and (iii) the control of nephrotic syndrome with apheresis [31].

Several factors have been mentioned, such as suPAR, cardiotrophin-like cytokine-1 (CLCF-1), apolipoprotein A1, anti-tyrosine phosphatase antibody of the O receptor, CAsK, and sCD40L [4,8,9,10,11,12,13,14], with different mechanisms of action. It seems obvious that the pathophysiology of FSGS is multifactorial. In order to prevent recurrence of FSGS on the kidney transplant, rituximab and apheresis (IA and plasma exchange (PLEX)) are the most frequent treatment used. Apheresis would make it possible to eliminate the soluble hyperpermeability factor, whereas rituximab (anti-CD20 monoclonal antibody) would have two mechanisms of action: (i) depletion of B lymphocytes and facilitating the production of regulatory T cells, thereby influencing the production of circulating factor; and (ii) inhibition of the degradation of actin in podocytes [32,33] by regulating the activity of acid sphingomyelinase using SMPDL-3B (sphingomyelin phosphodiesterase acid-like 3b).

None of the preventive intervention measures showed any benefits in recurrence of primary FSGS after kidney transplantation. However, Kalliopi Vallianou et al. [19] found that 90% of patients without recurrence had received prophylaxis by plasmapheresis, versus 62% of recurrent patients (*p* = 0.029). In contrast, Alasfar et al. [24] carried out a retrospective study that included 37 kidney-transplant recipients at high risk of recurrence for FSGS and that had received preventive treatment with PLEX and/or rituximab. A total of 23 (62%) of the 37 patients that received preventive treatment developed recurrence, compared to 14 (51%) recurrences in the 27 patients that received no treatment (*p* = 0.21). Likewise, Verghese et al. [25] retrospectively reviewed pediatric patients with FSGS (*n* = 57) and that had received a kidney transplant. They compared two groups (group 1: kidney transplant recipients after 2006 and that had received a PLEX before transplantation (*n* = 31); and group 2: kidney-transplant patients that received a transplant before 2006 and had not received a PLEX (*n* = 26)). They found no significant difference in the incidence (27 vs. 26%, *p* = 1.0) or the time until FSGS recurrence (*p* = 0.22) between the two groups.

Table 3 summarizes the main studies published for adults and children regarding prevention of FSGS recurrence after kidney transplantation by apheresis and/or rituximab. These were mainly retrospective studies. We found no prospective randomized studies. The preventive protocol varied from one study to the other. However, apart from the case reports, we noticed that prophylaxis by apheresis and/or rituximab did not prevent FSGS recurrence. Finally, genetic studies were also missing in most of the studies.

Plasmapheresis allowed complete or partial remission in 70% of children and 63% of adults when commenced soon after a recurrence [6]. Indeed, Trachtman et al. [46], in a literature review, reported that rituximab was associated with remission of nephrotic syndrome in ~75% of patients after FSGS recurrence on a kidney allograft. Likewise, Kashgary et al. [47] published a meta-analysis that included 413 renal-transplant recipients that had relapsing primary FSGS. After a median follow-up of 19 months, they found complete or partial remission in 71% (95% CI 66–75%) of patients after treatment with plasma exchange. Patients treated within 2 weeks of recurrence showed a trend towards a greater likelihood of remission (OR 2.16; 95% CI 0.93–5.01). In a study conducted by Kalliopi Vallianou et al. [19], patients with FSGS recurrence on a kidney allograft were treated by plasmapheresis and/or rituximab: this resulted in complete remission in 27% of cases and partial remission in 42.3% after an average therapy duration of 3 ± 1.79 and 4.4 ± 2.25 months, respectively.

In addition to plasmapheresis, semispecific IA has been shown to be effective at causing remission. Lionaki et al. [48] reported on 12 adult renal-transplant patients with recurrent FSGS that were treated with IA ± rituximab: after a mean follow-up of 48.3 months, there was complete remission in 58.3% of cases, and partial remission in 41.7%. Likewise, Allard et al. [49] reported complete remission in 67% and partial remission in 33% of 12 children that had undergone renal transplantation and had FSGS recurrence treated with IA sessions. After 3 months of IA treatment, two patients maintained remission without IA, and eight became IA-dependent [49].

To assess the benefit of adding rituximab to plasmapheresis to treat a FSGS relapse after kidney transplantation, Lanaret et al. [50] identified 148 adult FSGS patients that received a renal transplant between 2004 and 2018; of these, 109 received plasmapheresis (G1) and 39 received combined plasmapheresis with rituximab (G2). In the G1 group, rituximab was introduced only after failure of plasma-exchange therapy (*n* = 31); i.e., after an average of 28 days. Complete remission was achieved in 46.6% of patients, and partial remission was achieved in 33.1%. Analysis of the propensity score showed no difference in the rates of complete remission and partial remission between G1 (82.6%) and G2 (71.8%) (*p* = 0.08). Following the addition of rituximab, 26.3% of patients had complete remission, and 31.6% had partial remission. The incidence of severe infection was similar between patients treated with or without rituximab. In multivariate analysis, infectious episodes were associated with hypogammaglobulinemia < 5 g/L.

Our study had some limitations, including its relatively small sample size, retrospective nature, heterogeneity of treatments given, and absence of genetic testing. Indeed, only one of our patients underwent genetic exploration.

## 5. Conclusions

We concluded that pretransplant prophylaxis with plasmapheresis and rituximab did not appear to reduce the risk of recurrence of primary FSGS on the graft, but potentially could allow remission in the event of recurrence, to be confirmed by randomized studies. Because recurrence can occur several years after transplantation, this justifies rigorous monitoring of patients with FSGS, more so than for other kidney-transplant recipients.

## Figures and Tables

**Table 1:** Demographic data from the study population.

	**Patients (*n* = 17)**	FSGS **Recurrence: Group 1 (*n* = 8)**	No **Recurrence: Group 2 (*n* = 9)**	***p*-Value**
Age (years)	53 ± 11	47 ± 11	58 ± 9	0.04
Gender (M/F)	8/9	4/4	4/5	0.81
Age at FSGS diagnosis (years, min–max)	25 (4–55)	16 (4–55)	34 (6–48)	0.12
Time to progression to CKD5 (years, min–max)	5 (1–26)	2 (1–26)	6 (1–22)	0.99
Time to post-transplant recurrence (days, min–max)	3 (1–4745)	3 (1–4745)	NA	
Number of kidney transplants	1 (1–3)	2 (1–3)	1 (1–3)	0.13
Type of donor: – Brain death – Cardiac death – Living donor	11 4 2	6 0 2	5 4 0	0.109
Proteinuria at recurrence (g/L)	4.9 ± 3.6	4.9 ± 3.6		
Serum protein at recurrence (g/L)	61 ± 10	61 ± 10		
Serum creatinine at recurrence (µmol/L)	354 ± 235	354 ± 235		
eGFR at recurrence (mL/min/1.73 m²)	20 (4–77)	20 (4–77)		
Rituximab pre-renal transplant	8	7	1	0.001
Rituximab post-renal transplant	8	8	0	0.001
Number of rituximab doses at post-renal transplant	3 (2–5)	3 (2–5)	0	
IA or PE at pre-renal transplant	8	7	1	0.001
IA or PE at post-renal transplant	8	7	1	0.001
Number of PEs at post-renal transplant	20 (1–27)	20 (1–27)	8	
Number of IAs at post-renal transplant	60 (16–69)	60 (16–69)	0	
Average follow-up (years)	7 ± 4	7 ± 3	7 ± 4	0.98
AT1RB (yes)	14	7	7	1

Abbreviations: PE, plasma exchange; IA, immunoadsorption; M, male; F, female; eGFR, estimated glomerular-filtration rate; NA, not applicable; NS, nonsignificant; AT1RB, angiotensin-II type 1 receptor blocker.

**Table 2:** Characteristics of patients.

	**FSGS Recurrence: Group 1**								
**Patient**	**1**	**2**	**3**	**4**	**5**	**6**		**7**	**8**
Age at FSGS diagnosis (years)	4	12	17	9	18	15		55	28
Number of kidney transplants	1	1	3	3	3	3		1	2
Time to post-transplant recurrence (days)	3	3	1	4745	3	180		2	3
Proteinuria at recurrence (g/L)	3.85	3.13	4	6.73	1.28	3.35		4	13
Latest proteinuria (g/L)	0.06	NA	1.65	NA	2.62	0.3		3.67	2.8
Serum creatinine at recurrence (µmol/L)	110	277	350	142	760	235		553	401
Induction treatment	ATG	ATG	ATG	Basiliximab	ATG	ATG		ATG	ATG
Latest Serum creatinine (µmol/L)	107	Dialysis	76	Dialysis	108	202		286	138
Prophylaxis of recurrence before kidney transplantation	PE + rituximab	PE + rituximab	PE + rituximab	PE + rituximab	PE + rituximab	PE + rituximab			PE + rituximab
Treatment of recurrence	PE/IA + rituximab	rituximab	PE/IA + rituximab	PE + rituximab	PE/IA + rituximab	PE/IA + rituximab		PE/IA + rituximab	PE/IA + rituximab
	**No Recurrence: Group 2**								
**Patient**	**1**	**2**	**3**	**4**	**5**	**6**	**7**	**8**	**9**
Age at FSGS diagnosis (years)	34	44	25	48	44	35	6	16	26
Number of previous kidney transplants	1	1	1	1	1	1	3	2	2
Induction treatement	ATG	ATG	ATG	Basiliximab	ATG	ATG	ATG	Basiliximab	ATG
Latest serum creatinine (µmol/L)	71	58	120	131	78	152	131	168	81
Prophylaxis of recurrence before kidney transplantation	NA	NA	NA	NA	NA	NA	NA	NA	IA + rituximab
Prophylaxis of recurrence after kidney transplantation	NA	NA	NA	PE	NA	NA	NA	NA	NA

Abbreviations: PE, plasma exchange; IA, immunoadsorption; ATG, antithymocyte globulins; NA, not applicable.

**Table 3:** Studies evaluating apheresis and anti-CD20 in prevention of FSGS recurrence.

References	Types of Studies	Number of Patients	Age at KTx (Years)	Preemptive Protocol		Genetic Testing	IS (Induction, Maintenance)	Recurrence Rate	Graft Survival	Follow-Up Duration (Months)
				Apheresis	Anti-CD20					
Iguchi [34]	R	11	33 (20–43)	3 sessions PE or DFPP within 3 days before KTx in 3 patients	No	NA	ATG Cs, CsA, AZA	1/3 (33%) vs. 4/8 (50%)	100% vs. 50%	25
Ohta [35]	R	21	5.8 ± 3	2–3 sessions PE immediately before KTx in 15 patients	No	N/A	Cs, CsA/Tac, AZA/mizolibine	5/15 (33.3%) vs. 4/6 (66.7%)	87% vs. 60%	48 (7–127)
Gohh RY [36]	P	10	35 ± 12 (9–46)	8 sessions PE over a 2-week period before KTx	No	N/A	Basiliximab/ATG Cs, Tac, MMF	Three patients had recurrence of proteinuria (4–10 g/24 h) with FSGS at biopsy	Serum creatinine 111 ± 55 µmol/L Two patients with recurrence lost allograft function and returned to dialysis after 225 and 962 days	25 ± 10
Couloures K [37]	CR	1	18	4 sessions PE every other day before KTx and 6 sessions after KTx	No	Genetic testing for the mutations in the NPHS2 gene was negative.	Dacluzimab Cs, CsA, MMF	No	Serum creatinine 106 µmol/L	18
Fornoni [32]	R	41	15 ± 5.5 with RTX and 12.3 ± 5.2 without RTX	No	One dose of RTX (375 mg/m^2^) within 24 h of KTx in 27 patients	N/A	ATG/daclizumab/alemtuzumab Cs, Tac, MMF	7/27 (26%) vs. 9/14 (64%)	95.8% vs. 85.7% (*p* = 0.26)	12
Gonzalez [38]	R	34	13 ± 5	1–10 sessions PE before KTx in 17 patients	No	NPHS2 mutation testing on 10 patients (9 tested negative, 1 tested positive)	ATG/daclizumab Cs, CsA/Tac, MMF	9/17 (53%) vs. 10/17 (59%)	Graft loss: 25% in recurrence group vs. 20% in nonrecurrence group	36
Chikamoto H [39]	CR	1	7.5	4 sessions PE from days −12 to −5 before KTx	RTX 21 days before the KTx	Genetic testing for the mutations in the NPHS2 gene was negative	Basiliximab Cs, Tac, MMF	No	Serum creatinine 61 µmol/L	36
Audard V [33]	CR	4	36 (28–43)	No	RTX D0 in 2 patients and D0 and D7 in 2 patients	N/A	Basiliximab/ATG Cs, Tac/CsA, MMF	No	100% graft survival	25 (12–54)
Park [40]	R	27	39 ± 14	1 session PE before KTx in 4 cases 1 session PE with RTX before KTx in 5 cases	RTX was administered at 1 week before KTx	N/A	Basiliximab Cs, CsA/Tac, MMF	2/9 (22%) vs. 5/18 (27%) *p* > 0.05	FSGS with recurrence showed poor long-term graft survival, compared to without recurrence (*p* = 0.01)	49 (18–138)
Alasfar [24]	P	64	38 ± 16.5	3–10 sessions PE before KTx in 28 patients	One perioperative RTX (375 mg/m^2^) in 37 patients	N/A	ATG Cs, CsA/Tac, MMF	23/37 (62%) vs. 14/27 (51%): *p* = 0.21	100% no recurrence vs. 68% reccurence	80
Verghese [25]	R	57 = 31 before 2006 + 26 after 2006	13.2 ± 4.5 (after 2006) vs. 10.4 ± 5.4 (before 2006)	After 2006: 3 sessions PE before KTx with living donor and 1 session PE before KTx with deceased donor In postop for all patients after 2006: 5 sessions of PE every other day	One patient received two doses of RTX following PE	NPHS2 mutation testing negative	ATG Cs, CsA/Tac, MMF/AZA	7/26 (27%) vs. 8/31 (26%) *p* = 1.0	Death-censored graft survival was not significantly different (*p* = 0.61) and time to recurrence was not significantly different between both cohorts of patients (*p* = 0.22)	N/A
Sannomiya A [41]	R	5	33 (24–41)	LDL-apheresis: 1–2 sessions before KT	RTX 4 days before KT	N/A	Basiliximab Cs, Tac, MMF	No	Serum creatinine 111 (76–143) µmol/L	14 (2–22)
Katrin Kienzl-Wagner [42]	CR	1	5	No	Ofatumumab 175 mg/m^2^ in week 1, followed by 1150 mg/m^2^ weekly for five infusions	N/A	Basiliximab Cs, CsA, MMF	Yes, treated by 15 PE sessions and ofatumumab	Serum creatinine 32.3 µmol/L	8
Campise [43]	R	21	41 (38–52)	10 patients (2003–2008): 9 sessions PE for 3 weeks after KTx 11 patients (2008–2014): 1 session PE before KTx and 9 sessions after KTx	No	N/A	Basiliximab Cs, Tac, MMF	3/10 (30%) vs. 5/11 (45%) *p* = 0.65	3-year death-censored graft survival: 70% vs. 91% with *p* = 0.31	45 (30–107)
Uffing [44]	R	176	38 (29–47)	Prophylactic apheresis in 22 patients	No	N/A	ATG/basiliximab/daclizumab Cs, Tac/CsA, MMF	9/22 (41%) vs. 48/154 (31%)	Graft failure occurred in 18 patients (15%) without recurrence and in 22 patients with recurrence (39%)	78
Auñón [45]	R	34	32.9 ± 15.8	No	RTX, 1 g at induction and 1 g on day 14 after KT in 12 patients at risk for recurrence	Patients suspected of having had genetic or secondary forms of FSGS were excluded	ATG/basiliximab Cs, Tac, MMF	6/12 (50%) vs. 9/22 (40.9%)	53.5% graft loss with recurrence vs. 88.5% in nonrecurrence	71.7

## Data Availability

Data are available upon request.
